# Human health risk assessment of polycyclic aromatic hydrocarbons (PAHs) in smoked fish species from markets in Southern Nigeria

**DOI:** 10.1016/j.toxrep.2016.12.006

**Published:** 2016-12-30

**Authors:** Isioma Tongo, Ozekeke Ogbeide, Lawrence Ezemonye

**Affiliations:** Laboratory of Ecotoxicology and Environmental Forensics, Department of Animal and Environmental Biology, Faculty of Life Sciences, University of Benin, Benin City, Nigeria

**Keywords:** PAH, Smoked fish, Human health risk, Risk indices

## Abstract

Polycyclic Aromatic Hydrocarbons (PAHs) levels in four commonly consumed smoked fish species from markets in Southern Nigeria were assessed to evaluate possible human health risks associated with consumption. Varying levels of PAH congeners were observed in the fish tissues with the highest total concentration of PAHs in *Scomber scombrus*. High concentrations of benzo(a)pyrene was observed in *Clarias gariepinus* and *Ethmalosa fimbriata* with values above the guideline value of 0.05 mg/kg. The Dietary Daily Intake (DDI) value for total PAHs (∑PAHs) was highest for *S. scombrus* while the DDI value for the total carcinogenic PAHs (∑CPAHs) was highest for *E. fimbriata*. Carcinogenic human health risk assessment using carcinogenic toxic equivalents (TEQ), indicated that consumption of *E. fimbriata* has a higher potential to cause carcinogenic risks. TEQ values for all the fish species were however, below the estimated screening value (SV) of 3.556 mg/kg, while the estimated cumulative excess cancer risk (ECR) for *E. fimbriata* and *C. gariepinus* and PAH4 index for all the assessed fish species exceeded threshold values indicating potential carcinogenic risk from consumption.

## 1 Introduction

Polycyclic aromatic hydrocarbons (PAHs) are an important group of compounds of major environmental concern. There are several possible sources of PAHs in the environment, anthropogenic activities are, however, considered major sources of PAHs in the environment. Among the anthropogenic sources, petrogenic and pyrolytic sources are considered to be the most important. More than 100 PAHs have been characterized, 16 of which were classified by United States Environmental Protection Agency (USEPA) as priority pollutants because of their toxicity [1]. PAHs has been reported to be highly mutagenic and carcinogenic in humans [2].

Dietary exposures are the major source of human exposure to PAHs [3]. PAHs are found in food as a result of food processing techniques like curing, drying, smoking, roasting, grilling, barbecuing and refining. These food processing steps are known to generate and increase the level of PAHs in the food [4]. One significant food source of PAHs is smoked fish [5]. In developing countries, smoked fish is a common source of protein in most diets, smoke not only gives the fish special taste and aroma, it also improves preservation due to its dehydrating and bactericidal properties [5], [6]. However, smoke especially wood smoke contains PAHs, many of which are carcinogenic [3]. In developing countries, smoking is the most common method employed in preserving fish. In Nigeria, smoked fish products constitute about 61% of the total 194,000 m t of dry fish produced [6]. Smoked fish products are the most available form of fish product for consumption [6], which could be attributed to the fact that most of the fishing communities have limited access to electricity to preserve their fish products [6]. This has however increased the risk of PAHs contamination through consumption. Food Safety is of growing concern globally and PAHs residues if present in smoked fish above recommended levels could pose serious public health concerns [7]. Previous studies have shown the presence of PAHs in smoked fish [3], [8], [5], [6], however, studies on human health risk associated with consuming smoked fish are rather scanty [9], especially for reported studies from Nigeria.

The objective of this study was, therefore, to determine the concentration of PAHs in four locally available and commonly consumed smoked fish species (*Clarias gariepinus*, *Ethmalosa fimbriata*, *Tilapia zilli*, and *Scomber scombrus*) from markets in Southern, Nigeria, in order to assess possible human health risks associated with consumption.

## 2 Materials and methods

### 2.1 Sample collection

**Fig. 1:**
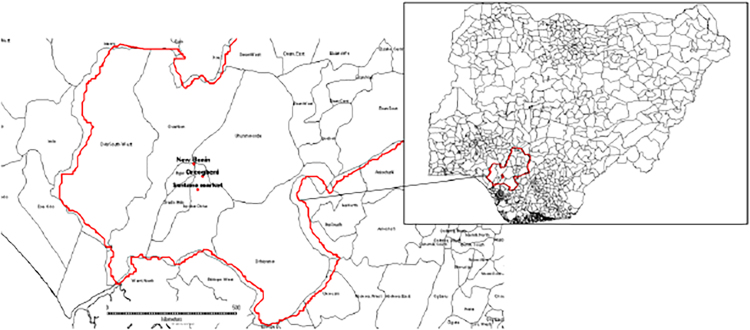
Map of Study area showing the major markets.

Samples of *Clarias gariepinus*, *Tilapia zilli*, *Ethmalosa fimbriata*, and *Scomber scombrus*, were randomly collected from three major markets (Oreogbeni (6°21′ 0.09″N and 5° 39′ 32.67″E), New Benin (6°23′ 59.96″N and 5° 36′ 67.37″E) and Santana market (6°17′ 44.6″N and 5° 38′ 8.9″E) in Southern, Nigeria (Fig. 1).

The selected markets are major sources of smoked fish for most markets in Southern Nigeria. Samples were wrapped in aluminum foil, packed in labeled polythene bags and transported to the laboratory for analysis. Samples were collected every month for four months (June–September).

### 2.2 Extraction and analysis

Extraction of PAHs was carried out based on the method described by [10]. 10 g of the homogenized fish sample was thoroughly mixed with anhydrous Na_2_SO_4_ to dehydrate the sample. 20 ml of the extraction solvent (di-chloromethane) was added to the sample. Samples were covered with aluminum foil to prevent evaporation and sonicated to separate supernatants of extracts. Extracts were concentrated using an evaporator. Extracts were then cleaned up using a chromatographic column, moderately packed at the bottom with 1 cm glass wool. 2 g of silica gel and 1 cm of anhydrous Na_2_SO_4_ was added to the column while the column was pre-eluted with 20 ml dichloromethane. Extracts were then concentrated and collected in 2 ml vials.

#### 2.2.1 Chromatographic analysis

The cleaned up extracts were analysed for naphthalene, acenaphthylene, benzo[b]fluoranthene, phenanthrene, dibenzo[a,h]anthracene, chrysene, benzo[a]pyrene, acenaphthene, benzo[k]fluoranthene, fluorene, pyrene, benzo[a]anthracene, anthracene, fluoranthene, indeno[1,2,3-cd]pyrene, and benzo[g,h,i]perylene. Corresponding results were obtained using Gas chromatography (GC, Hewlett-Packard HP-5890 Series II with flame ionization detection (GC-FID)). The GC was programmed as follows: initial temperature of 60 °C for 2 min and ramped at 25 °C/min to 300 °C for 5 min and allowed to stay for 15 min giving a total of run time of 22 mins. A 2 μL volume splitless injection mode was used and the injection port temperature was set at 250 °C, while 300 °C was maintained for the injection port of the FID detector. A standard mixture of 17 priority PAHs (Naphthalene, Acenaphthylene, Acenaphthene, Fluorene, Phenanthrene, Anthracene, Fluoranthene, Pyrene, Benzo(a)anthracene, Chrysene, Benzo(k)fluoranthene, Benzo(a)pyrene, Benzo(b)fluoranthene, Indeno(1,2,3) perylene, Dibenzo(a,h)anthracene and Benzo(g,h,i) perylene) was obtained and used for the analysis. Compounds were identified by comparing the retention time of standards with that obtained from the extracts and individual analysis of PAHs were used for quantitation.

Naphthalene, Acenaphthylene, Acenaphthene, Fluorene, Phenanthrene, Anthracene, Fluoranthene, Pyrene were the assessed non-carcinogenic PAHs, while Benzo(a)anthracene, Chrysene, Benzo(k)fluoranthene, Benzo(a)pyrene, Benzo(b)fluoranthene, Indeno(1,2,3) perylene, Dibenzo(a,h)anthracene and Benzo(g,h,i) perylene) were the assessed carcinogenic PAHs [1].

### 2.3 Human health risk estimations

#### 2.3.1 Regulatory limits and guidelines

The toxicological risk associated with PAH concentrations in smoked fish was assessed through comparison of the observed concentrations with regulatory limits and guidelines. Concentration of PAHs in the smoked fish species were assessed for individual PAH concentrations, total PAH concentrations (sum of all the assessed PAH congeners) and total carcinogenic PAHs (sum of the carcinogenic PAHs [1], namely Benzo(a)anthracene, Chrysene, Benzo(k)fluoranthene, Benzo(a)pyrene, Benzo(b)fluoranthene, Indeno(1,2,3) perylene, Dibenzo(a,h)anthracene). Concentrations of Benzo(a)pyrene (B(a)P), which has been accepted as a marker for the occurrence and effect of carcinogenic PAHs in smoked foods as specified in the European Commission Regulation(EC) No 1881/2006,was compared with the maximum acceptable level of 0.005 mg/kg [11] for benzo[α]pyrene in smoked fish.

#### 2.3.2 Potential human health risk from consumption of smoked fish

To assess human health risks from exposure to PAHs through consumption of smoked fish (dietary intake), human intake models were applied. The Dietary Daily Intake (DDI) concentrations of PAH’s from consumption of contaminated smoke fish species were assessed. Carcinogenic risks were also assessed by evaluating the carcinogenic potencies of individual PAH concentrations(B(A)Pteq), the Carcinogenic toxic equivalents (TEQs) and the Excess Cancer Risk Index. Values used for parameterization of the human intake models is presented in Table 1.

**Table 1:** Human Intake Model Parameters.

Parameters	Unit	Value	Reference
Concentration of each congener (*Ci*)	mg/kg	Table 2	Table 2
Fish ingestion rate *(IFR)*	Kg/capita/day	0.0548	[19]
Toxicity equivalence factor (*TEFi*)	No Unit	[12]	[12]
Carcinogenic potency of Benzo[a]Pyrene (*Q)*	mg kg^−1^ d^−^	7.3 mg/kg/d	[13]
Exposure Duration (*ED)*	years	30	[14]
Adult body weight (*BW*)	kg	70	[15]
average life span *(ATn)*	days	8760	[16]
Concentration of Benzo(a)anthracene *(B[a]A)*	mg/kg	Table 2	Table 2
Concentration of Chrysene *(Chr)*	mg/kg	Table 2	Table 2
Concentration of Benzo(a)pyrene *(B[a]P)*	mg/kg	Table 2	Table 2
Maximum acceptable risk level *(RL)*	dimensionless	10^−5^	[17]
Oral Slope Factor *(SF)*	mg/kg/day	[1]	[1]
Reference Dose (RfD)	mg/kg/day	Table 3	[1]

##### 2.3.2.1 The dietary daily intake (DDI)

The Dietary Daily Intake (DDI) of PAH’s in the smoke fish species was assessed for adult population using Eq. (1) [18]. The daily intake of PAHs from smoked fish was evaluated by multiplying the respective PAH concentration in each fish sample by the fish ingestion rate *(IFR*) of an average weight adult (70 kg) from Nigeria. The consumption rated for fish in Nigeria for an average adult population was obtained from data of the Food Agriculture Organization (FAO) 2014 [19], on Fishery and aquaculture statistics. Evaluation of Dietary Daily Intake (DDI) was calculated for individual PAH congers, the sum of the 16 PAHs analyzed (Total PAHs) and also for the sum of those PAHs considered possible human carcinogens (Total Carcinogenic PAHs).

(1) The Dietary Daily Intake(DDI)=Ci×IFR

##### 2.3.2.2 Carcinogenic risk assessment of PAHs in smoked fish

Cancer risk due to dietary exposure to PAHs in smoked fish was assessed using the PAH4 index, Individual PAH carcinogenic potencies, the carcinogenic toxic equivalents (TEQs) and the excess cancer risk index.

The PAH4 index was assessed in this study based on the of review by the Contaminants in the Food Chain (CONTAM) Panel, in 2008 relating to occurrence and toxicity of PAHs in food, which concluded that PAH4 is a more suitable indicators of PAHs in Food [20]. PAH4 was evaluated using Eq. (2), as the sum of four different polycyclic aromatic hydrocarbons, namely benzo[a]anthracene (B[a]A), chrysene (Chr), benzo[b]fluoranthene (B[b]FL), and benzo[a]pyrene (B[a]P). The estimated PAH4 index of each fish species was then compared with the maximum permissible level to determine the occurrence and effect of carcinogenic PAHs in the smoked fish samples. The maximum permissible level of 0.03 mg/kg for the sum of PAH4 in smoked fishery products as recommended by the European Union (EU) Commission Regulation, No 1327/2014 in 2014 [11], as regards maximum levels of polycyclic aromatic hydrocarbons (PAHs) in traditionally smoked fish and fishery products was applied.

(2) PAH4 Index (PAH4) = ∑(B[a]A + Chr + B[b]FL + B[a]P)

The Carcinogenic potencies of individual PAHs B(A)Pteq was evaluated by multiplying the PAH concentration in the sample by the individual toxicity equivalency factor (TEF) (Eq. (3)) [12]. The TEF is an estimate of the relative toxicity of individual PAH fraction compared to benzo(a)pyrene. Toxic equivalency factors have been applied as a useful tool for the regulation of compounds with a common mechanism of actions (e.g PAHs).The TEFs developed by Nisbet and LaGoy [12] was applied (Table 3) and these values were used to calculate PAH as benzo[a]pyrene equivalents for a standard adult with 70 kg body weight.

(3) Carcinogenic potencies of individual PAHs (B(A)P*teq*) = Ci × TEF*i*

**Table 2:** Mean concentration (mg/kg) of PAH on smoked fish species from markets in Southern, Nigeria.

	PAH (mg/kg)	CODE	*Clarias gariepinus*		*Tilapia zilli*		*Ethmalosa fimbriata*		Scomber scombrus	
			MEAN	SD	MEAN	SD	MEAN	SD	MEAN	SD
*	Naphthalene	NaP	0.056	0.11	0.315	0.38	0.003	0.00	1.171	0.318
*	Acenaphthylene	AcPY	0.044	0.02	0.000	0.00	0.058	0.03	0.489	0.371
*	Acenaphthene	AcP	0.055	0.06	0.052	0.03	0.032	0.04	0.535	0.635
*	Fluorene	Flu	0.013	0.01	0.000	0.00	0.019	0.02	0.420	0.393
*	Phenanthrene	Phe	0.045	0.03	0.069	0.05	0.067	0.03	0.100	0.091
*	Anthracene	Ant	0.029	0.03	0.215	0.25	0.039	0.03	0.306	0.345
*	Fluoranthene	FL	0.012	0.01	0.030	0.06	0.008	0.01	0.176	0.157
*	Pyrene	Pyr	0.015	0.03	0.048	0.07	0.001	0.00	0.187	0.149
***	Benzo(a)anthracene	BaA	0.012	0.02	0.001	0.00	0.000	0.00	0.076	0.067
***	Chrysene	Chr	0.135	0.27	0.220	0.38	0.047	0.09	0.078	0.073
**	Benzo(k)fluoranthene	BkFL	0.000	0.00	0.000	0.00	0.000	0.00	0.040	0.060
***	Benzo(a)pyrene	BaP	0.204	0.20	0.000	0.00	0.288	0.23	0.007	0.013
***	Benzo(b)fluoranthene	BbFL	0.095	0.09	0.000	0.00	0.132	0.11	0.000	0.00
**	Indeno(1,2,3)pyrene	Ind	0.000	0.00	0.000	0.00	0.000	0.00	0.000	0.00
**	Dibenzo(a,h)anthracene	DBA	0.000	0.00	0.000	0.00	0.000	0.00	0.000	0.00
**	Benzo(g,h,i,)perylene	BP	0.000	0.00	0.000	0.00	0.000	0.00	0.000	0.00
	Total PAH	∑PAH	0.715	0.34	0.951	0.76	0.694	0.36	3.585	0.80
	Total Carcinogenic PAH	∑CPAHs	0.446	0.23	0.222	0.38	0.467	0.28	0.201	0.13

* Non-Carcinogenic PAHs.

** Carcinogenic PAHs.

*** Carcinogenic PAH and PAH used to derive the PAH4 Index.

**Table 3:** Estimated Dietary daily intake (DDI), Carcinogenic potencies (B(A)Pteq), and Excess cancer risk (ECR) of PAHs in smoked fish species from markets in Southern, Nigeria.

PAH		RfD	TEF	*Clarias gariepinus*			*Tilapia zilli*			*Ethmalosa fimbriata*			*Scomber scombrus*		
				DDI	B(A)Pteq	ECR	DDI	B(A)Pteq	ECR	DDI	B(A)Pteq	ECR	DDI	B(A)Pteq	ECR
				(mg/day)	(mg/kg)	(mg/kg)	(mg/day)	(mg/kg)	(mg/kg)	(mg/day)	(mg/kg)	(mg/kg)	(mg/day)	(mg/kg)	(mg/kg)
*	NaP	0.02	0.001	3.05E-03	5.57E-05	1.09E-09	1.73E-02	3.15E-04	6.17E-09	1.62E-04	2.96E-06	5.79E-11	6.42E-02	1.17E-03	2.29E-08
*	AcPY	NA	0.001	2.41E-03	4.41E-05	8.62E-10	0.00E + 00	0.00E + 00	0.00E + 00	3.16E-03	5.76E-05	1.13E-09	2.68E-02	4.89E-04	9.56E-09
*	AcP	0.06	0.001	3.03E-03	5.53E-05	1.08E-09	2.84E-03	5.18E-05	1.01E-09	1.77E-03	3.23E-05	6.32E-10	2.93E-02	5.35E-04	1.05E-08
*	Flu	0.04	0.001	7.37E-04	1.35E-05	2.63E-10	0.00E + 00	0.00E + 00	0.00E + 00	1.04E-03	1.90E-05	3.72E-10	2.30E-02	4.20E-04	8.23E-09
*	Phe	NA	0.001	2.46E-03	4.48E-05	8.77E-10	3.78E-03	6.90E-05	1.35E-09	3.68E-03	6.72E-05	1.31E-09	5.47E-03	9.98E-05	1.95E-09
*	Ant	0.3	0.01	1.57E-03	2.87E-04	5.61E-09	1.18E-02	2.15E-03	4.21E-08	2.15E-03	3.91E-04	7.66E-09	1.68E-02	3.06E-03	5.99E-08
*	FL	0.04	0.001	6.48E-04	1.18E-05	2.31E-10	1.66E-03	3.03E-05	5.92E-10	4.35E-04	7.94E-06	1.55E-10	9.67E-03	1.76E-04	3.45E-09
*	Pyr	0.03	0.001	8.13E-04	1.48E-05	2.90E-10	2.63E-03	4.80E-05	9.39E-10	7.86E-05	1.43E-06	2.81E-11	1.02E-02	1.87E-04	3.65E-09
***	BaA	NA	0.1	6.50E-04	1.19E-03	2.32E-08	6.85E-05	1.25E-04	2.45E-09	0.00E + 00	0.00E + 00	0.00E + 00	4.18E-03	7.63E-03	1.49E-07
***	Chr	NA	0.01	7.43E-03	1.35E-03	2.65E-08	1.21E-02	2.20E-03	4.31E-08	2.57E-03	4.69E-04	9.17E-09	4.27E-03	7.78E-04	1.52E-08
**	BkFL	NA	0.1	0.00E + 00	0.00E + 00	0.00E + 00	0.00E + 00	0.00E + 00	0.00E + 00	0.00E + 00	0.00E + 00	0.00E + 00	2.19E-03	3.99E-03	7.82E-08
***	BbFL	NA	1	1.12E-02	2.04E-01	3.99E-06	0.00E + 00	0.00E + 00	0.00E + 00	1.58E-02	2.88E-01	5.63E-06	3.68E-04	6.72E-03	1.32E-07
***	BaP	NA	0.1	5.20E-03	9.48E-03	1.86E-07	0.00E + 00	0.00E + 00	0.00E + 00	7.24E-03	1.32E-02	2.58E-07	0.00E + 00	0.00E + 00	0.00E + 00
**	Ind	NA	0.1	0.00E + 00	0.00E + 00	0.00E + 00	0.00E + 00	0.00E + 00	0.00E + 00	0.00E + 00	0.00E + 00	0.00E + 00	0.00E + 00	0.00E + 00	0.00E + 00
**	DBA	NA	5	0.00E + 00	0.00E + 00	0.00E + 00	0.00E + 00	0.00E + 00	0.00E + 00	0.00E + 00	0.00E + 00	0.00E + 00	0.00E + 00	0.00E + 00	0.00E + 00
**	BP	NA	0.01	0.00E + 00	0.00E + 00	0.00E + 00	0.00E + 00	0.00E + 00	0.00E + 00	0.00E + 00	0.00E + 00	0.00E + 00	0.00E + 00	0.00E + 00	0.00E + 00

TEF values for the PAHs was adopted from [12].

* Non-Carcinogenic PAHs.

** Carcinogenic PAHs.

*** Carcinogenic PAH and PAH used to derive the PAH4 Index.

The carcinogenic toxic equivalents (TEQs) was then obtained by summing the carcinogenic potencies of individual PAHs (B(A)Pteq) [13] (Eq. (4)).

(4) Carcinogenic toxic equivalents (TEQs) = ∑B(A)P*teq*

The evaluated TEQ value was compared with a Screening value to assess the health risks of PAHs to humans from fish consumption. The screening value (SV) is the threshold concentration of chemicals in edible tissue that is of potential public health concern [21], [22]. The screening value was calculated using Eq. (5) [21], [22].

(5) $$Screening\;Value\;(SV)-\frac{\left[\left(RL/SF\right)XBW\right]}{IFR}$$

The excess cancer risk induced by dietary exposure to PAHs via smoked fish consumption was assessed using Eq. (6) [23].

(6) $$Excess\;Cancer\;Risk\;(ECR)=\frac{\sum^{\;}Q\;\;X\;\;\text{B}\;\;(\text{A})\;\;\text{P}teq\;\;\;X\;\;\;IFR\;\;\;X\;\;\;ED}{\left(BW\;\;\;X\;\;\;ATn\right)}$$

### 2.4 Statistical analysis

Data analysis were performed using Microsoft Excel 7.0 program. Multiple bar graphs were used for the pictorial description of assessment endpoints. Individual PAHs, Total PAHs (∑PAHs) and total carcinogenic PAHs (∑CPAHs) concentrations were summarized separately for each fish species using descriptive statistics (means, range, standard deviation, standard error). Statistical differences between individual PAH concentrations, low and high molecular weight PAHs, ring types, dietary daily intake (DDI), and carcinogenic potencies of individual PAH concentrations (B(A)Pteq), between the species were performed using Analysis of variance (ANOVA) at 0.05 level of significance.

## 3 Results and discussion

### 3.1 Concentration of PAHs in smoked fish

The mean concentrations of individual PAH congeners in the smoked fish species samples from the various markets in Southern Nigeria is presented in Table 2. The total concentrations (mg/kg) of PAHs were 0.715, 0.951, 0.694, and 3.585 in *C. gariepinus*, *T. zilli, E. fimbriata,* and *S. scombrus* respectively. The high values of PAHs observed in the smoked fish samples might be attributed to the smoking process during preparation and preservation. The basic process of fish smoking in Nigeria is by heating the fish over partially burning wood. This process generates wood smoke which gives the fish special taste and aroma, however, most PAHs in smoked foods, especially fish, comes from wood smoke [8] and wood smoke has been reported to contain a large number of PAHs [24]. The total carcinogenic PAH concentrations (mg/kg) were 0.446, 0.222, 0.467, and 0.201 in *C. gariepinus*, *T. zilli, E. fimbriata,* and *S. scombrus* respectively (Table 2).

Total mean concentrations of PAHs was highest in *S. scombrus* (Table 2), with mean concentrations of individual PAHs ranging from 0 to 1.172 mg/kg. Similar results of higher concentrations were reported for *S. scombrus* by [25]; in a study of PAHs concentration in six species of smoked fish from in Western Nigeria. Mean concentrations (mg/kg) of PAH congeners in the other fish species ranged from 0 to 0.204 (*C. gariepinus),* 0–0.315 (*T. zilli*), and 0–0.288 (*E. fimbriata).* PAH concentrations between the fish species were significantly different (p < 0.05, F = 4.26).The differences observed in PAHs concentration between the species could be attributed to differences in fat and moisture compositions of each species alongside the nature of the skin cover [26]. Results from this study is similar to studies by Igwe et al. [25], Yusuf et al. [27] and Silva et al. [6], who reported varying concentrations of PAHs in different species of smoked fish in Nigeria. Total PAH in smoked fish species observed in this study was also compared with PAH in smoked fish observed from other parts of Nigeria [6], [25], [28], [27] and other countries [29], [30], [5]. It was observed that concentrations in this study were above these reported concentrations. This may be attributed to the increased use of the traditional smoking method in the preparation and preservation of fish. Several studies have shown that smoking fish using traditional methods, increases the amount of PAH formed in smoked fishes [6], [27]. Naphthalene was the most dominant congener in *T. zilli,* and *S. scombrus,* making up 33.3%, and 32.7% of the total PAH residues respectively (Table 2), while Benzo(b)fluoranthene was the most dominant congener in *C. gariepinus* and *E. fimbriata* comprising 28.5% and 41.5% respectively (Table 2).

The total carcinogenic PAH concentrations were highest in *E. fimbriata* (0.467 mg/kg), although concentrations were not significantly different (p > 0.05, F = 0.42) between the species. This high concentrations observed in these species may possibly be linked to the type of smoking process used during the preparation of this fish species. Pyrolysis of polycyclic aromatic hydrocarbon residues leads to the formation of additional higher molecular weight polycyclic aromatic hydrocarbons and, consequently increases the polycyclic aromatic hydrocarbon concentration in the samples [31]. *E. fimbriata* is usually prepared using an intense smoking process that involves the direct exposure to hot smoke from burning wood fire a process that takes place for about 2–5 days [32]. The high concentration of total carcinogenic PAHs in *E. fimbriata* is, therefore, a course for concern.

Benzo(a)pyrene concentrations were observed to be highest in *C. gariepinus* and *E. fimbriata* with mean concentrations of 0.10 and 0.13 mg/kg respectively. Observed concentrations were higher than the maximum acceptable level (0.005 mg/kg), for benzo[α]pyrene in smoked fish [33] which calls for serious health concern. Considerable attention has been drawn to B(a)P residues in foods because of its possible carcinogenicity to humans [34], [35]. However, Benzo(a)pyrene concentrations were only observed in 6% of all the assessed smoked fish sample.

### 3.2 Human health risk assessment of PAH in smoked fish

#### 3.2.1 Dietary daily intake (DDI) of PAH’s from consumption of smoked fish

In Nigeria, there is a high rate of consumption of fishes and smoked fish products are the most ready form of fish product for consumption [36]. Therefore, using the concept of DDI to assess the health risk of toxicants is imperative. The concept of DDI to assess health risks from toxicants is usually applied because of differences in fish consumption rates [37]. Table 3 summarizes the dietary daily intake (DDI) of PAH’s in the analysed smoke fish samples for an adult (70 kg) population in southern Nigeria. DDI values (mg/day) estimated from individual PAH concentrations in smoked fish ranged from 0 to 0.0112 (*C. gariepinus),* 0–0.0173 (*T. zilli),* 0–0.0158 (*E. fimbriata,*), *and.* 0–0.0642 (*S. scombrus*) (Table 3). Differences in DDI values between the fish species were statistically significant (p < 0.05, F = 4.26). The DDIs (mg/day) of Benzo(a)pyrene, which is one of the most potent animal carcinogen were 0.005 and 0.007 for *C. gariepinus* and *E. fimbriata* respectively. Benzo(a)pyrene was not detected in *T. zilli* and *E. fimbriata.* The DDI for *E. fimbriata* was again highest.

The estimated DDI (mg/day) values for total PAHs (∑PAHs) in the assessed smoked fish were 0.039, 0.052, 0.038, 0.195 for *C. gariepinus, T. zilli, E. fimbriata and. S. scombrus* respectively (Table 4), while the DDI (mg/kg) for the total carcinogenic PAHs were 0.025, 0.012, 0.026 and 0.011 for *C. gariepinus, T. zilli, E. fimbriata and. S. scombrus* respectively (Table 4). The DDI value for total PAHs (∑PAHs) was highest for *S. scombrus* while the DDI value for the total carcinogenic PAHs (∑CPAHs) was highest for *E. fimbriata*. The results indicate that the consumption of *S. scombrus* in preference to the other fish species will result in higher risk of exposure to PAHs while the consumption of *E. fimbriata* will result in higher risk of exposure to carcinogenic PAHs.

**Table 4:** Estimated Carcinogenic Risk Indices of PAHs in smoked fish species from markets in Southern, Nigeria.

Carcinogenic Risk Index	*Clarias gariepinus*	*Tilapia zilli*	*Ethmalosa fimbriata*	*Scomber scombrus*
∑DDI (mg/kg)	0.03917	0.0521011	0.038051079	0.196471657
∑ DDI for Carcinogenic PAH (mg/kg)	0.02445	0.0121382	0.025582025	0.011002397
TEQ	0.21643	0.0049918	0.302145	0.025260718
PAH4 (mg/kg)	0.446	0.222	0.467	0.161
SV	3.556	3.556	3.556	3.556

A comparison with other studies showed that the observed DDI values for ∑CPAHs and ∑PAHs through the consumption of these fish species were higher than reported studies from Ghana [37] with DDI values of 201 ng/d and 7548 ng/d for ∑CPAHs and ∑PAHs respectively. DDI values for ∑PAHss in the fish species were also higher than reported studies from Spain with DDI values of 626–712 ng/d [38], India with DDI values of 1.77–10.7 ng/kg bw/day [39] and Kuwait with DDI values of 231 ng/d [40]. Higher DDI values observed in this study could be attributed to the relatively higher concentrations of PAHs in the smoked fish species assessed in this study.

The higher values obtained in this study may also be attributed to the higher use of the traditional method for processing and preserving these fish which might have invariably caused an increase in PAH concentrations in these fish species. Fish vendors in Nigeria usually re-smoke fish continuously until they are sold in the market in order to increase the shelf life of the fish [6], since preservation using electricity is usually not feasible. The DDI's for individual PAH concentrations were also compared to the available reference dose [1] to determine long-term risk from exposure to PAHs residues through the consumption of smoked fish. The observed DDI values were however, generally below the reference dose for all the smoked fish species assessed.

#### 3.2.2 Carcinogenic risk assessment of PAHs in smoked fish

The estimated PAH4, individual PAH carcinogenic potencies, carcinogenic toxic equivalents, screening value and excess cancer risk are presented in Table 3, Table 4.

##### 3.2.2.1 PAH4

The values of PAH4 observed in smoked fish species were 0.45, 0.22, 0.26, 0.47, 0.16 mg/kg for *C. gariepinus, T. zilli, E. fimbriata, and S. scombrus* respectively (Table 4). These observed values were above the recommended limits (0.03 mg/kg) set by the European Union [20], for PAHs in smoked fish and smoked fishery products. The result, therefore, implies that consumption of these fish species could pose potential health effects to humans.

This result is similar with PAH4 values reported by Iwegbue et al. [41] for *C. gariepinus* (0.255 mg/kg)*, E. fimbriata* (0.185 mg/kg) *and S. scombrus* (0.0151 mg/kg), however PAH4 values from this study were slightly higher. This can be attributed to the relatively higher concentrations of PAHs observed in these smoked fish species.

##### 3.2.2.2 Individual PAH carcinogenic potencies (B(A)Pteq)

Individual PAH carcinogenic potencies varied among the smoked fish species assessed, differences in B(A)Pteq values between the species were however not statistically different (p > 0.05, F = 0.57). Benzo(b)fluoranthene had the highest carcinogenic potency (mg/kg) in *C. gariepinus* (0.20) and *E. fimbriata* (0.29), *Chrysene in T. zilli* (0.002), and Benzo(a)anthracene in *S. scombrus* (0.008) (Table 3). These values were higher than what was observed for smoked fish species from Western Nigeria [27]. Individual PAH carcinogenic potencies in the assessed smoked fish samples were also below the maximum acceptable level of 0.005 mg/kg for benzo[α]pyrene in smoked fish [33]. However values for Benzo(b)fluoranthene were observed to be above the maximum acceptable level except in *T. zilli* (Table 3).

##### 3.2.2.3 Carcinogenic toxic equivalents (TEQ) of PAHs in the smoked fish

The TEQ approach was implemented to directly assess the carcinogenicity of PAH contamination of the smoked fish species [42]. The carcinogenic toxic equivalents (TEQ) of PAHs in the fish species were 0.22, 0.005. 0.30, 0.03 mg/kg in *C. gariepinus*, *T. zilli, E. fimbriata,* and *S. scombrus* respectively (Table 4). The result indicates that *E. fimbriata* has a higher potential to cause carcinogenic risks from consumption than the other species even when the total mean concentrations of PAHs was lowest compared to the other species. This can be attributed to the way *E. fimbriata* is processed. *E. fimbriata*, also known as Bonga fish is usually prepared by smoke-drying for 2–5 days over very high fire from burning wood. The longer the firing process the longer the fish will keep, hence very high temperature is employed in the smoking process [32]. Wood smoke contains a large number of PAHs [8] whose formation is basically dependent on temperature. The temperature of smoke is important because the amount of PAHs formed during pyrolysis increase as the smoking temperature increases [2]. High-temperature smoking increases PAH concentrations to high levels and may contain a large variety of PAHs, including the most carcinogenic ones [2]. TEQ values reported in this study were higher than values reported by Yusuf et al., [27], for PAH in smoked fish from Western Nigeria, especially for Catfish. Furthermore, the observed TEQ values for the assesses smoked fish values were also higher than values reported by Iwegbue et al. [41] for *C. gariepinus* (0.06 mg/kg)*, E. fimbriata* (0.0057 mg/kg) *and S. scombrus* (0.00258 mg/kg) collected from markets in Nigeria.

##### 3.2.2.4 Screening value of PAHs in the smoked fish

The screening value (SV) was evaluated to assess the health risks of PAHs to humans from consuming these four fish species. This value is the threshold concentration of a chemical in edible tissues that is of potential public health concern [21], [22]. An estimated screening value (SV) of 3.556 mg/kg was obtained. Results showed that the TEQ values for all the fish species were below the SV of 3.556 mg/kg (Table 4). Results from this study, were not consistent with the report of Ikue et al. [43], in a study of PAH contaminants in *Chrysichthys nigrodidatatu* in Rivers State, Nigeria, who reported calculated TEQ values above estimated screening values, indicating potential health effects. Nozar et al. [44] also reported higher TEQ values when compared to calculated SV values for PAH in seafood (fish, crab, and bivalve) in Iran at a consumption rate of 55.1 g/d. However, results from this study agrees with reported studies of PAH concentrations in fish (feral finfish) from Hong-Kong market [21], and PAHs in the common eel (*Anguilla anguilla*) from River Tiber, Italy [45] were the estimated TEQ values were lower than the SV.

##### 3.2.2.5 Excess cancer risk and PAH4 index of PAHs in the smoked fish

The excess cancer risk (ECR) of an adult population with an average weight of 70 kg caused by dietary exposure to PAHs was evaluated. The estimated ECR resulting from lifetime exposure to PAHs through fish consumption were compared to the acceptable guideline value of 10^−6^ set by USEPA [46]. USEPA stipulates that a level of risk where there is a lifetime cancer risk of one in a million (ECR = 10^−6^) over a 70 year lifetime period, is considered acceptable, while an instance where there is an additional lifetime cancer risk of one in ten thousand or greater (ECR = 10^−4^), is considered serious [54].

In this study, the ECR values for all the assed PAH congeners in all the fish species were lower than the USEPA threshold value (10^−6^) (Table 3). However, cumulative excess cancer risk for *E. fimbriata* (0.00000591 mg/kg) and *C. gariepinus* (0.00000424 mg/kg) exceeded the USEPA’s acceptable cancer risk level of 10^−6^ having higher cancer estimates than the other smoked fish species. This indicates that consumption of *E. fimbriata* and *C. gariepinus* in preference to the other species of fish could result in potential cancer risk.

Similar studies in India [39] and Ghana [37] have reported estimated excess cancer risk from consumption of fish above the USEPA guideline values. In addition, similar studies on excess cancer risk from consumption of other foods has also been reported above the guideline values [47], [48]

## 4 Conclusion

Priority polycyclic aromatic hydrocarbon (PAH) were detected in four species of smoked fish obtained from major markets in Southern Nigeria. Varying levels of PAH congeners were observed in the smoked fish species with the highest total concentration of PAH in *S. scombrus.* The high concentration of benzo(a)pyrene in *C. gariepinus and E. fimbriata* above the guideline value of 0.05 mg/kg calls for serious health concern. Furthermore, the observed values of PAH4 and the cumulative ECR for *E. fimbriata* and *C. gariepinus* above the recommended values is also a cause of worry. The study, therefore, deduces that there are substantial exposure and possible carcinogenic human health risk from consumption of smoked fish species from Southern Nigeria. Education of fish mongers and vendors on safer processing and preserving alternatives is imperative.
